# Notch activation enhances mesenchymal stem cell sheet osteogenic potential by inhibition of cellular senescence

**DOI:** 10.1038/cddis.2017.2

**Published:** 2017-02-02

**Authors:** Ye Tian, Ying Xu, Taiyang Xue, Longgang Chen, Bin Shi, Bing Shu, Chao Xie, Massimo Max Morandi, Todd Jaeblon, John V Marymont, Yufeng Dong

**Affiliations:** 1 Department of Orthopedics, Shengjing Hospital, China Medical University, Shenyang, China; 2 Department of Anesthesiology, Shengjing Hospital, China Medical University, Shenyang, China; 3 Department of Orthopedics, Spine Research Institute, Longhua Hospital, Shanghai University of Traditional Chinese Medicine, Shanghai, China; 4 Department of Orthopedics, University of Rochester Medical Center, Rochester, NY, USA; 5 Department of Orthopedic Surgery, Louisiana State University Health Sciences Center, Shreveport, LA, USA

## Abstract

Our previous studies have confirmed the therapeutic effects of mesenchymal stem cell (MSC) monolayer sheet transplantation on allograft repair. A limiting factor in their application is the loss of MSC multi-potency as a result of high density sheet culture-induced senescence. In the study reported in this article, we tested whether Notch activation could be used to prevent or delay sheet culture-induced cell aging. Our results showed that, during *in vitro* long-term (5-day) cell sheet culture, MSCs progressively lose their progenitor characteristics. In contrast, Notch activation by Jagged1 in MSC sheet culture showed reduced cellular senescence and cell cycle arrest compared with control MSCs without Notch activation. Importantly, knockdown of Notch target gene Hes1 totally blocked the inhibition effect of Jagged1 on cellular senescence. Finally, the *in vivo* allograft transplantation data showed a significant enhanced callus formation and biomechanical properties in Notch activation cultured long-term sheet groups when compared with long-term cultured sheet without Notch activation. Our results suggest that Notch activation by Jagged1 could be used to overcome the stem cell aging caused by high density sheet culture, thereby increasing the therapeutic potential of MSC sheets for tissue regeneration.

Mesenchymal stem cells (MSCs) provide a promising cell source for bone tissue regeneration, which is already under investigation in several clinical trials.^1, 2^ However, appropriate delivery and retention of MSCs or osteogenic cells during therapeutic strategies for skeletal repair remain extremely challenging. While conventional *in vivo* delivery methods (single-cell suspensions, injection, seeding to or mixed with scaffolds) have demonstrated some success,^3, 4, 5, 6^ problems still remain, including rapid cell diffusion, uneven cell distribution across the scaffolds, and weak adhesion of MSCs seeded to the scaffolds, causing easy detachment from graft following *in vivo* transplantation. To overcome these problems, stem cell sheets generated in temperature-responsive culture dishes have been used by our group to enhance allograft healing during repair of large bone defects.^7^ Since cell sheet culture often induces stem cell aging, characterized by increased senescence and a rise in the levels of the cell cycle inhibitors p16 and p21,^8, 9^ only short-term (1-day) cultured stem cell monolayer sheets have been tested in our mouse allograft healing model. Although these short-term cultured cell sheets showed a significant increase of bone callus formation around allografts, studies on MSC maintenance in prolonged cell sheet culture are still essential in developing ready-to-use cell sheets for clinical application.

MSC sheet-based applications to skeletal regeneration and repair in the clinic would require large numbers of functional cells and high density cell sheet culture often induces rapid stem cell aging process accompanied by loss of proliferative and differentiation capacity. To maintain stem cell phenotype in cultures and avoid cell sheet culture-induced cell aging has become necessary.^10^ Various signaling factors have been implicated in the regulation of MSC maintenance and expansion, including FGF, Wnt and TGF pathways. Recently, we identified the Notch signaling pathway as an important regulator of MSC proliferation and differentiation using an *in vivo* mouse model^11^ and successfully utilized Jagged1 (JAG1)-mediated Notch activation to rapidly expand MSCs in regular cell cultures.^12^ Here we undertook a study to further determine if Notch signaling could be used to prevent or delay rapid cell aging induced by long-term (5-day) sheet culture. In addition, prolonged culture provides extra time for surgeon to make flexible transplantation schedule, which avoids any unnecessary MSC discarding.

The Notch signaling pathway is an evolutionarily conserved signaling system that regulates cell proliferation, differentiation, and fate determination in both embryonic and adult organs. In mammals, Notch signaling is initiated when one of the 11 Notch ligands (Jagged1-2; DLL1,3,4; DLK1-2; MAGP1-2; DNER; and NB3) activates a single-pass transmembrane cell surface Notch receptor (Notch1-4), resulting in a series of receptor cleavage events (S2 and S3) that release the Notch intracellular domain (NICD) into the cytoplasm via ADAM and Presenilin (PSEN1/2) protease activities. NICD translocates to the nucleus and binds the transcriptional regulators, RBPj*κ* and MAML, creating a transcriptionally active complex. Upon activation, NICD-RBPj*κ*-MAML ternary complexes drive the expression of downstream target genes, such as the Hes and Hey family of bHLH transcription factors.^11, 13, 14^ This axis of classic Notch signaling is often referred to as the RBPj*κ*-dependent canonical Notch pathway. Studies of Notch function in embryonic stem cells, somite progenitors, and neural progenitors have demonstrated that ultradian oscillations of Notch and Hes signals are required for the normal development and maintenance of various cell lineages and tissues.^15, 16^ Our previous study identified JAG1-Notch2-Hes1 as the dominant Notch molecules in MSCs,^12^ we further tested whether the Notch ligand JAG1 could be utilized to inhibit senescence in long-term sheet culture, and finally enhance their therapeutic effect *in vivo*.

## Results

### Increased MSC aging in response to cell sheet culture

**Figure 1:**
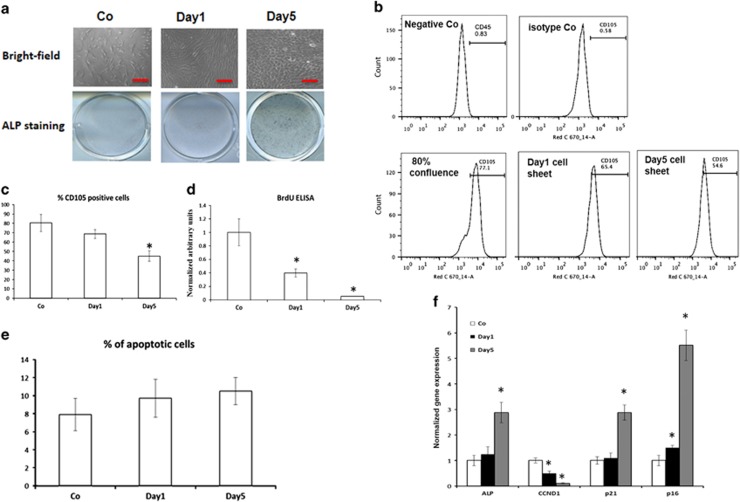
Cell sheet culture induces differentiation in mesenchymal stem cells (MSCs). (**a**) The effect of sheet culture on MSC morphologic change was examined in bright field (upper). Scale bar represents 100 *μ*m. ALP staining revealed MSC differentiation (lower). (**b**) Representative flow cytometry histograms showing CD105 expression in MSCs from control (80% confluence), day1 cell sheet, and day5 cell sheet. (**c**) Quantification of the CD105 subpopulations in total MSCs from flow cytometry data. (**d**) BrdU ELISA to monitor proliferation rate in MSCs from control (Co) (80% confluence), day1 cell sheet (Day1), and day5 cell sheet (Day5). (**e**) The percent of apoptotic cells was measured by Caspase-3/7 assay in MSCs from control (80% confluence), day1 sheet, and day5 sheet. (**f**) Gene expression for MSC differentiation marker (ALP), cell cycle regulator (CCND1, p16, p21) in control, day1 sheet, and day5 sheet was measured by quantitative polymerase chain reaction. Results are mean±S.D. All experiments were performed in triplicate. *P<0.05 *versus* control cells

While long-term culture sometime is needed in clinical settings. However, cell aging and differentiation can be accelerated due to the high degree of cell–cell contact in long-term culture.^17, 18^ To study the effects of cell sheet culture on MSC aging, we compared MSCs from 1-day and 5-day sheets with control MSCs from 80% confluence culture. Cell morphology changes were first observed in a bright field. Control and 1-day sheet MSCs showed a similar fibroblast-like appearance with few cytoplasmic expansions, while MSCs from 5-day sheets were found to be significantly larger and widely spread out (Figure 1a, upper panel). These findings suggest sheet-culture induces cell differentiation and senescence in MSC culture. The enhanced cell differentiation was further confirmed by alkaline phosphatase (ALP) staining, which showed increased ALP activity in 5-day MSC sheets compared with control and 1-day MSC sheets (Figure 1a, lower panel).

The effects of biologic aging on immunophenotypic change of MSCs were analyzed by flow cytometry for cell surface antigen CD105, a typical marker for less differentiated stromal cells. The percentage of CD105 positive cells was 79.8±8.6% in 80% confluence MSCs, 70.7±3.5% in 1-day sheet MSCs, and 45.5±5.2% in 5-day sheet MSCs (Figures 1b and c) suggesting a progressive loss of stromal cell population in cell sheet cultures. To eliminate lymphocyte contamination, lymphocyte cell surface marker CD45 was also analyzed and <1% of the cells were positive for this marker. In addition, the appropriately matched isotype control (lgG-FITC) was used to set the gate for the CD105-positive population (Figure 1b).

To assess sheet culture-dependent changes in MSC proliferation, we performed a BrdU-labeling based cellular proliferation assay. MSCs from the 5-day sheet culture exhibited a markedly reduced proliferation rate compared with control MSCs and 1-day sheet cultures (Figure 1d). Interestingly, cell apoptosis was not found to be significantly increased in 5-day sheet culture compared with control and 1-day cultures when assayed by caspase 3/7 activity (Figure 1e) indicating that apoptosis was not involved in this aging process. To further validate this age-related alteration in cell sheet cultures, markers associated with the aging phenotype of stromal cells (e.g., the cell-cycle regulation protein *cyclinD1, p16, and p21*) were analyzed by real-time PCR (Figure 1f). As expected, cell proliferation marker *cyclinD1 (CCND1)* expression was significantly reduced in 5-day cultures compared with control and 1-day cultures, whereas the expression of cell cycle inhibitors *p16* and *p21* was significantly up-regulated in 5-day sheet cultures. Consistent with ALP staining, the RNA expression of MSC spontaneous differentiation marker ALP was also significantly induced in 5-day cultures compared with control and 1-day cultures, further confirming progressive cell aging differentiation in long-term cell sheet cultures.

### Decreased MSC senescence within JAG1-coated plates

**Figure 2:**
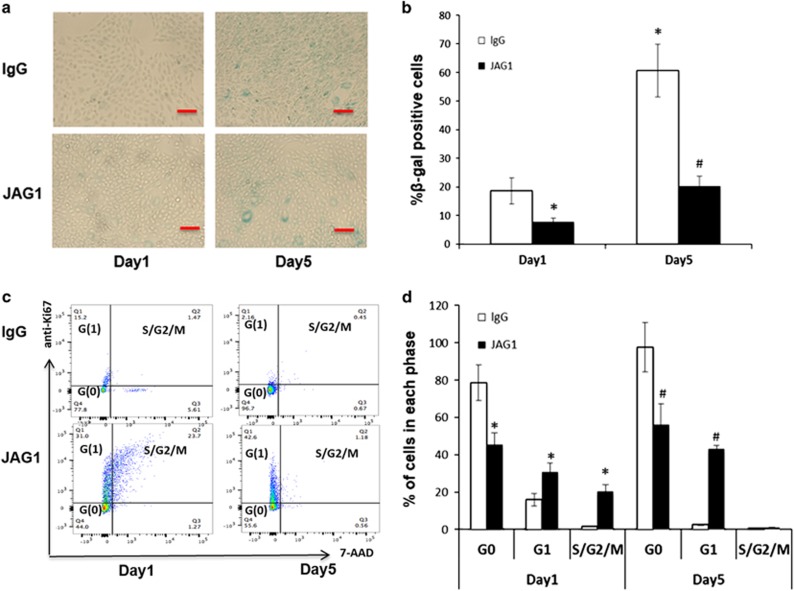
The effects of JAG1 on MSC senescence and cell cycle distribution in sheet cultures. (**a**) SA-*β*-gal staining was performed to determine the extent of senescence in 1- and 5-day sheet cultures with JAG1 or lgG treatment. Scale bar represents 100 *μ*m. (**b**) SA-*β*-gal positive cells were quantitated by ImageJ. (**c**) Representative flow cytometry graphs of cell cycle analysis. **(d**) The percentage of cells in G0, G1, and S/G2/M from 1- and 5-day sheet cultures. Results are mean±S.D. All experiments were performed in triplicate. *P<0.05 *versus* lgG day1 control cells; ^#^*P*<0.05 *versus* lgG day5 control cells

Cellular senescence is a phenomenon of cell aging, which can be detected by monitoring senescence associated *β*-galactosidase activity (SA-*β*-gal).^17^ To monitor the senescence status of MSCs during sheet culture, we performed *β*-galactosidase staining in 1-day and 5-day cultures. Our data showed that the percentage of MSCs positive for SA-*β*-gal increased with time, from 19.25±4.6% at day 1 to 60.48±8.29% at day 5 (Figures 2a and b), indicating a progressive cell senescence in long-term sheet cultures. Since our previous study showed that Notch activation by JAG1 enhances stem cell maintenances *in vivo* and *in vitro*, we further monitored cellular senescence in long-term sheet culture on JAG1 ligand coated plates. As we expected, activation of Notch signaling by JAG1 led to a reduced cell senescence in both 1- and 5-day sheet cultures, as demonstrated by decreased frequencies of SA-*β*-gal positive cells (8.25±1.5% and 20.5±4.2%, respectively) when compared with MSCs from lgG control groups (*P*<0.05) (Figures 2a and b).

Cheung and Rando reported that cell cycle regulation is required for MSCs to remain in an undifferentiated state and to exit the cell cycle during cellular senescence progression.^19^ To further determine the effect of JAG1 on cell cycle distribution, we performed flow cytometric analysis using MSCs from 1-day and 5-day sheet cultures after labeling them with 7-AAD and Ki67. The percentages of cells in the G0, G1, and S/G2/M phases of the cell cycle were then calculated. In lgG control groups, MSCs in 1-day and 5-day cultures possessed a high percentage of cells in the G0 phase (80.96±5.5% and 97.5±5.4%, respectively) due to sheet culture-induced cell cycle arrest. By comparison, the G0 phase cells in JAG1 groups was 45.2±2.8% and 57.5±8.2% in the 1-day and 5-day cultures respectively, and significantly lower than those in the lgG groups (Figures 2c and d). These data demonstrate an increased fraction of cells arrested in the G0 phase during long-term sheet cultures. Collectively, these studies indicate that Notch activation could reduce senescence progression by promoting more cells into G1 and S/G2/M phases, and supporting our hypothesis that activation of Notch signaling delays cell aging in sheet cultures.

**Figure 3:**
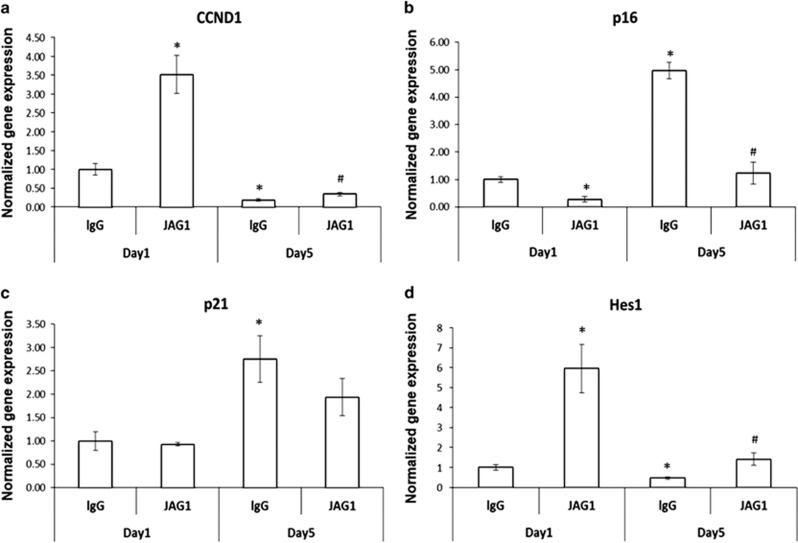
mRNA expression profile of MSC changes in cell sheet cultures. Differential expression of cell-cycle-related genes was verified by quantitative RT-PCR. (**a**) CCND1 gene expression increased by JAG1 in 1-day cell sheet cultures. (**b**) p16 gene expression decreased by JAG1 in both 1- and 5-day cell sheet cultures. (**c**) p21 gene expression increased from day 1 to day 5 in control cell sheet cultures but was not altered by JAG1 treatment. (**d**) Hes1 gene expression decreased from day 1 to day 5 in control cell sheet cultures, and JAG1 significantly induced Hes1 expression in both 1- and 5-day cultures. Results are mean±S.D. All experiments were performed in triplicate. *P<0.05 *versus* lgG day-1 control cells; ^#^*P*<0.05 *versus* lgG day5 control cells

To obtain insight into the anti-aging effects of JAG1 in MSCs at the molecular level, we monitored the expression of the proliferation marker CCND1 and the typical senescence markers, p16 and p21 in both short-term and long-term sheet cultures. Consistent with our previous finding, *CCND1* expression was induced by JAG1 in 1-day cultures and no significant changes were observed in 5-day cultures compared with lgG controls (Figure 3a). In lgG control group, relative quantification by RT-PCR revealed an ~5-fold up-regulation of *p16* RNA (Figure 3b) and a 2.7-fold up-regulation of *p21* RNA in 5-day cultures compared with 1-day cultures (Figure 3c), which suggests that these two factors act synergistically to induce MSC senescence. Interestingly, although both p16 and p21 plays an important role in cellular senescence, only *p16* expression was significantly reduced and maintained in lower levels in the JAG1 groups at both 1- and 5-day cultures. In contrast, JAG1 did not show a significant inhibition effect on *p21* gene expression in both 1- and 5-day cultures (Figures 3b and c). These observations suggest that p16, not p21, is the major downstream factor responsible for the Notch-induced anti-aging effect in cell sheet cultures. Taken together, these results demonstrate that MSC aging induced by cell sheet culture can be remarkably reduced by activation of Notch signaling.

Since JAG1 effectively reversed MSC senescence in cell sheet culture, we next investigated the possible Notch signaling downstream molecules involved in this process. Previously we had determined that Notch signaling maintains and expands MSCs via a JAG1-Notch-Hes1 signaling axis during mouse skeletal development.^11^ Therefore, we further measured Notch target *Hes1* expression in 1- and 5-day sheet cultures. RT-PCR analysis of total RNA revealed a decreased expression of *Hes1* from 1-day to 5-day cultures in control lgG-coated plates, while *Hes1* expression at both time points was significantly increased by JAG1 (Figure 3d). This suggests that canonical Notch target gene Hes1 is involved in this JAG1-mediated inhibition of MSC aging.

### Knockdown of Hes1 expression blocks JAG1-mediated inhibition of MSC aging

**Figure 4:**
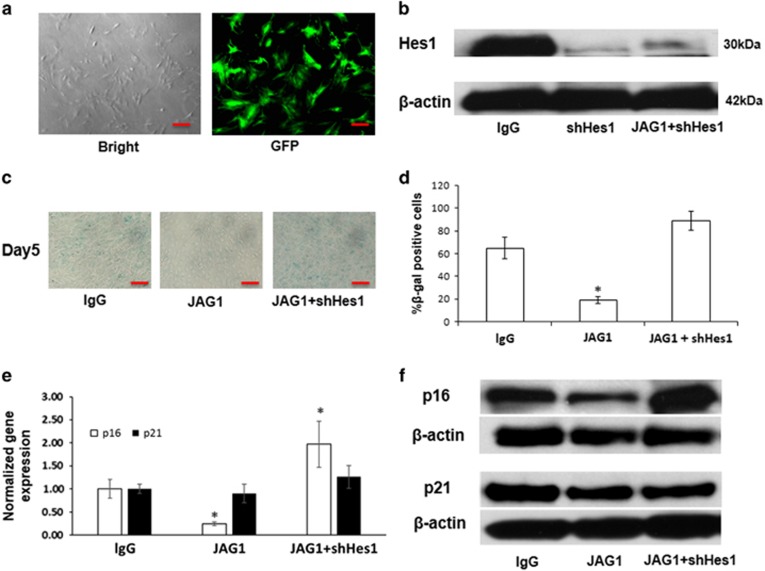
Hes1 is required for JAG1-induced inhibition of MSC agin. (**a**) Bright field image showed a high infection efficiency in lentiviral GFP control MSCs after 24 h of culture. Scale bar represents 50 *μ*m. (**b**) Western blot analysis confirmed the decrease of Hes1 protein levels at day 5 in Hes1 knockdown MSCs, even with JAG1 treatment. (**c**) SA-*β*-gal staining was performed to determine the extent of senescence in shHes1-infected MSCs at day 5 in cell sheet culture with JAG1 treatment. Scale bar represents 100 *μ*m. (**d**) SA-*β*-gal positive cells in cell sheet cultures were quantitated by ImageJ. (**e**) Quantitative reverse transcriptase polymerase chain reaction (qRT-PCR) data showed that the decreased expression of p16 by JAG1 was rescued by knockdown of Hes1 in MSCs at day5 in sheet cultures. No changes were observed in p21 expression in 5-day sheet cultures. (**f**) Western blot data showed a similar expression pattern of p16 and p21 protein level to RNA expression in JAG1 treated wild type MSCs and Hes1 deficient MSCs. Results are mean±S.D. All experiments were performed in triplicate. *P<0.05 *versus* lgG control cells

To further test whether Hes1 is required in JAG1-mediated inhibition of cellular senescence, we knocked down Hes1 expression using shRNA lentiviral particles in MSC sheet cultures. After 24 h of culture with lentivirus, GFP controls showed that 90% of MSCs were successfully infected by lentiviral particles (Figure 4a). Western blot analysis using protein from 5-day sheet cultures further confirmed these knockdown results by showing a significantly reduced Hes1 protein expression in shRNA lentivirus-infected MSCs with or without JAG1 treatment (Figure 4b). More importantly, although the cellular senescence in 5-day cultures was significantly inhibited by JAG1-mediated Notch activation, this inhibitory effect was almost completely abrogated after knocking down Hes1 expression in these MSCs (Figure 4c). Furthermore, a significantly increased number of SA-*β*-gal–positive cells in Hes1 shRNA-infected MSCs was observed and quantified by ImageJ (Figure 4d). Furthermore, real-time PCR data showed an increased gene expression of *p16*, but not *p21*, in Hes1 shRNA-infected MSCs (Figure 4e). Finally, protein expression of *p*16 in western blot confirmed our PCR results by showing thinner band in JAG1 treated cells and thicker band in Hes1 deficient cells, and no significant change was observed in protein expression of *p*12 (Figure 4f), demonstrating that *p16* is one of the most responsible factors for Notch activation-mediated MSC maintenance in cell sheet cultures. Overall, we have demonstrated that Notch inhibition of MSC aging is dependent on the presence of Notch canonical ligand JAG1 and target gene Hes1.

### MSC sheets increase bone callus formation and biomechanical properties of femoral allografts

**Figure 5:**
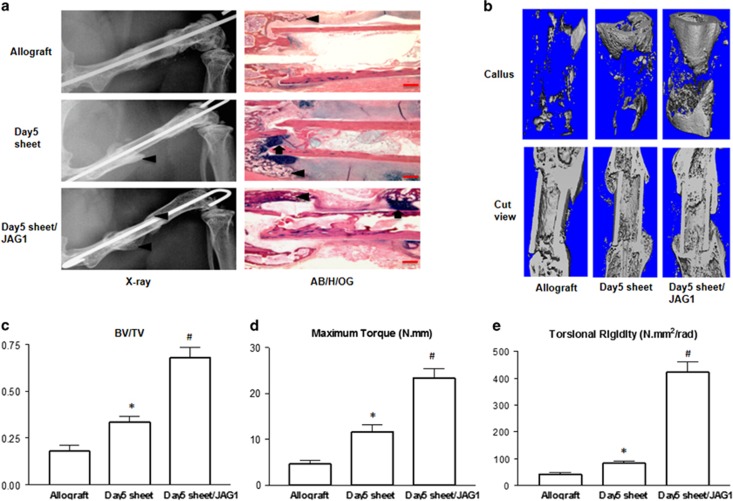
5-day MSC sheets enhance bone callus formation and improve the biomechanical torsional properties of grafted femurs at 6-weeks post-surgery. (**a**) The left panels are representative x-ray images of allografts alone, allografts wrapped with lgG cultured 5-day sheets (Day5 sheets), and allografts wrapped with Notch activation cultured 5-day MSC sheets (Day5 sheet/JAG1) at 6-weeks post-surgery. Right panels show the histological sections stained with AB/H/OG. Scale bars=400 *μ*m. Bone callus is marked with black triangles and cartilaginous callus is marked with black arrows. (**b**) Representative Micro-CT volumetric rendering of the grafted femurs with 5-day lgG or JAG1 MSC sheets at 6-weeks post-surgery. Allograft alone was used as the control. (**c**) Quantification of bone volume/total tissue volume (BV/TV) from the mineralized calluses of each group. (*, *P*<0.05 compared with allograft alone; ^#^, *P*<0.05 compared with allograft+Day5 sheets). (**d**) Maximal torque and (**e**) Torsional rigidity of grafted femurs from each group was retrieved upon mice (*n*=6) sacrifice at 6-weeks post-surgery. Data presented as mean±standard deviation. *P<0.05 compared with allograft alone; ^#^*P*<0.05 compared with allograft+Day5 sheets

Since we have previously studied the therapeutic effects of 1-day monolayer MSC sheets in a mouse allograft repair model, here we further test whether Notch activation-cultured long-term 5-day MSC sheets can also be used *in vivo* as a pseudo-periosteum to enhance allograft bone defect healing. In these experiments, we transplanted allografts alone, allograft wrapped with lgG-cultured 5-day MSC sheets, or allografts wrapped with Notch activation (JAG1)-cultured 5-day MSC sheets into our mouse femoral bone defect models. At six weeks post-surgery, x-ray images (Figure 5a left panel) showed a minimal amount of new callus was formed between the allograft and host bone in the allograft alone groups. lgG/5-day MSC sheet-wrapped allografts exhibited large bony callus formation around the proximal allograft and host bone junction, but no significant callus formation was ever observed near the distal allograft and host bone junction. In contrast, a large bridging callus was observed surrounding both ends of allograft in JAG1/5-day MSC-sheet group and the callus formed at proximal gap even reached the mid-allograft surface. AB/H/OG stained sections (Figure 5a right panel) confirmed that a large amount of cartilaginous soft callus were still observed at the host/allograft junction in lgG/5-day MSC-sheet groups at 6 weeks, while more bony callus and less cartilaginous soft callus were shown in JAG1/5-day MSC-sheet groups. This suggests a more mineralized bone formed by Notch activation.

To further confirm our X-ray and histological findings, we performed Micro-CT analysis using samples from 6-weeks post-surgery. Micro-CT analyses (Figure 5b) demonstrated that the external callus at the host/allograft junction in both MSC sheet groups contained more mineralized bone than the allograft alone groups. However, only JAG1/5-day sheet groups showed a large amount of bony callus formed in the distal end of the allograft gap. The percentage of bone volume (BV) to total tissue volume (TV) of callus was calculated, and identified as significantly higher in the JAG1/5-day MSC-sheet groups when compared to lgG/5-day sheet groups and allograft alone groups (Figure 5c). Finally, to determine whether increased bone callus formation could be translated into improved mechanical properties, we performed torsional testing on the grafted femurs at 6-weeks post-surgery. Our results showed increased biomechanical properties in lgG/5-day MSC sheet groups were observed when compared with allograft alone groups (Figures 5d and e). More importantly, the biomechanical strength in JAG1/5-day MSC sheet groups was even higher than that in lgG/5-day groups by showing maximum torque of 22.4 N mm and torsional rigidity of 442.2  N mm^2^/rad, respectively (Figures 5d and e).

## Discussion

For several years, cell sheet technology using temperature-responsive culture dishes has been applied to tissue engineering to regenerate damaged tissues.^20, 21^ As mentioned above, we recently demonstrated that tissue-engineered periosteum generated with short-term MSC sheet culture enhances callus formation during allograft repair.^7^ However, due to the often un-expected transplantation schedule in the clinical settings, long-term cultured MSC sheets should also be studied for their therapeutic efficacy. To monitor MSC maintenance in long-term sheet culture, we first measured the phenotypic change of MSCs in 1-day and 5-day cell sheet cultures. Consistent with previous findings, we observed a progressive cell aging from day 1 to day 5 by showing enhanced cell spontaneous differentiation and senescence. This is in line with cell cycle analysis that cell sheet culture stimulates MSCs cell cycle exit from G1 and S/G2/M phases to the G0 phase, which is an initial step for cell senescence.

As described earlier, senescence is a critical cellular response for continuous aging. Increased senescence often reduces cell proliferation and differentiation potential and impairs tissue regeneration. It is characterized by increased cell cycle arrest in the G0 phase, altered gene expression of growth regulatory proteins (such as p21 and p16),^22^ morphologic transformations, and enhanced senescence-associated SA-*β*-gal activity.^23^ In addition, culture senescence limits culture time, thereby preventing cell expansion to the numbers required for cell-based therapies.^10^ These observations pose a significant challenge that must be overcome in order to apply cellular therapies in clinical settings. Since both MSC maintenance and lineage differentiation are controlled extrinsically by cellular signals,^24^ one of the most promising approaches to prevent MSC *ex vivo* aging is to target the signaling pathways required for MSC maintenance. Published studies have demonstrated that several signaling pathways might be involved in cellular senescence. For example, Ras/Raf/MEK/ERK is reported to be activated in oncogene-induced senescence.^25^ Interestingly, our previous data showed that activation of Notch signaling by JAG1 in MSCs not only promotes cell proliferation but also inhibits cell differentiation to keep cells in a younger stage.^12^ These findings led us to test whether Notch signaling could be used to prevent or reverse MSC senescence induced by cell sheet culture. Data from this study clearly support our hypothesis by showing that JAG1-mediated Notch activation significantly reduced the number of senescence cells in long-term MSC sheet cultures. In addition, less MSCs in G0 phase were observed in JAG1-coated plates confirmed this JAG1 dependent anti-aging effect.

**Figure 6:**
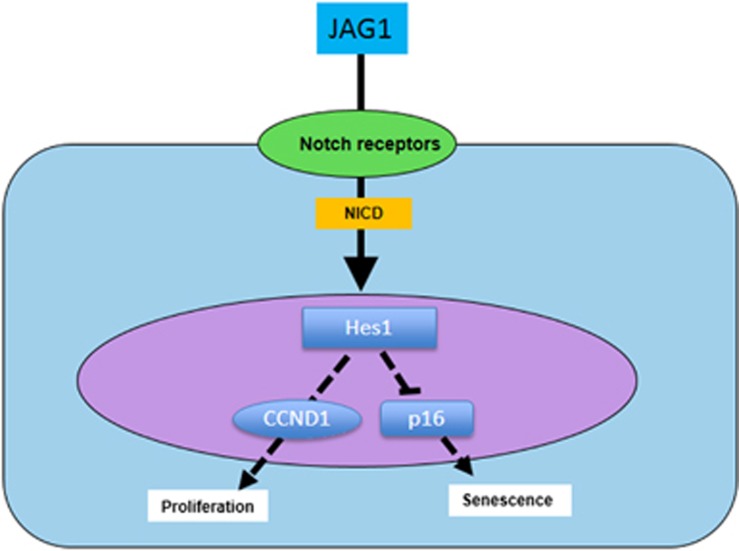
Possible model of Notch signaling transduction in regulation of MSC *in vitro* aging. Activations of Notch signaling by Jagged1 (JAG1) in MSC sheet culture stimulates cell proliferation and inhibits aging. Notch signaling includes sequential stimulation of a signaling cascade involving Notch intracellular domain (NICD) and Hes1. NICD induces the expression of Hes1, which may lead to activation of Cyclin D1 (CCND1) and inhibition of p16, leading to the delay of MSC aging in cultures

Rayess and associates reported that p16 expression is associated with the cellular senescence process through a telomere-dependent or telomere-independent mechanism in most mammalian tissues.^26^ With regard to cell cycle checkpoints, p16 controls the G1-S transition by binding to CDK4/6, inhibiting its kinase activity and thereby preventing Rb phosphorylation.^27^ Consistent with previous studies, our results further show that Notch activation by JAG1 reduces MSC cellular senescence and decreases expression of *p16*, but not of *p21*, in cell sheet cultures, suggesting that p16 plays an important role in JAG1-mediated inhibition of MSC senescence (Figure 6). Our cell cycle analysis further supported this conclusion by showing reduced G0 phase cell populations in cell sheet cultures with Notch activation by JAG1.

Although our data indicate that JAG1-mediated Notch signaling alone can partially reverse MSC aging in cell sheet culture, whether it functions through canonical or non-canonical Notch signaling is still unknown. Notch target genes have been identified in various cellular and developmental contexts,^13, 28^ including Hes1, Hes5, and Hes7, as well as Hey1, Hey2, and HeyL. As our previous study demonstrated that Hes1 is the most responsive factor in MSCs for JAG1-mediated Notch activation,^11^ we further examined the role of Hes1 in JAG1-mediated inhibition of MSC aging. Our loss-of-function experiments clearly show that knockdown of Hes1 expression in MSC sheet culture totally abrogates JAG1-mediated anti-aging effects, suggesting that Hes1 is the major molecular responsible for the inhibition of cell senescence (Figure 6). Further in-depth studies are needed to understand the mechanistic participation of Hes1-regulated anti-aging effects in cells, including how Hes1 regulates p16 and p21 activity in MSCs.

Since increased MSC senescence often impairs their therapeutic potential *in vivo*, we further examined the effects of Notch activation cultured 5-day sheets, which contains fewer senescent MSCs on allograft healing. Allograft alone and lgG-cultured 5-day MSC sheets that contain more aging senescence MSCs were used as controls. Because human MSCs show immune modulation effect and do not induce significant inflammatory immune response in C57BL/6 J mice, we decided to use wild type C57BL/6 J mice in this study to validate the effect of human stem cell sheet on allograft healing. As we expected, the results clearly showed a significantly increase of bone callus formation and biomechanical property in Notch activation cultured 5-day sheet groups indicating JAG1-coated plates culture could be utilized to enhance MSC osteogenic potential via inhibition of MSC aging. It should be noted that there were less bone callus formed in lgG-cultured 5-day sheet groups when compared with JAG1-cultured 5-day sheets, but it was significantly more than that found in allograft alone groups suggesting even aged MSC sheet can facilitate bone callus formation. One possible reason is that while the MSCs in long-term cultured sheets are aging, the extracellular matrix surrounding MSCs contains active growth factors that may attract or induce endogenous MSCs migration and differentiation towards osteoblasts *in vivo*.

## Conclusions

In summary, JAG1-mediated Notch activation was used in this study to prevent MSC aging in long-term sheet cultures and to enhance cell sheet osteogenic potential *in vivo*. Our study demonstrates that activation of Notch signaling is a promising novel strategy for reversing the aging clock in senescent MSCs and for promoting the appropriate application of Notch signaling to enhance the therapeutic effects of MSCs for regeneration of bone tissue.

## Materials and methods

### Animal study design

C57BL/6 J male mice were purchased from Jackson Laboratory. Allogeneic bone grafts were obtained from mice of the 129/J strain for implantation into C57BL/6 J mice. Louisiana State University Committee of Animal Resources approved all animal surgery procedures (Protocol # P-15-005). Experiments were designed to include 12 male mice samples per group. Host mice carrying allografts were randomly and equally assigned to either control (Allograft alone), 5-day lgG-coated plates cultured MSC-sheet or 5-day JAG1-coated plates cultured MSC-sheet groups. The sample size (*n*=6) for Micro-CT and biomechanical testing and the size (*n*=6) for histology was determined by power analysis based on our pilot experiment data.

### Cultivation of MSCs and lentivirus transduction

MSCs derived from human bone marrow were obtained from Lonza Group Ltd. (http://www.lonza.com). MSCs were first cultured in MSCGM Mesenchymal Stem Cell Growth Medium (Lonza, Cat. PT-3001) until 80% confluence as passage 1 (P1) cells. Further expanded P1 stem cells were harvested as P2 MSCs for different assays as described below. Hes1-specific (shHes1) and control (Co) short hairpin RNA (scrambled shRNA) lentiviral particles were purchased from Sigma-Aldrich. For lentiviral infection, 2000 cell/mm^2^ MSCs were seeded in six-well plates (Nunc, polystyrene) and incubated for 24 h at 37 °C prior to being combined with shRNA lentivirus in the presence of 8 *μ*g/ml polybrene (Sigma-Aldrich). The infected MSC cultures were harvested at day 5 after the cell sheet had formed. Protein expression of Hes1, cell cycle inhibitor p16 and p21 in cells was detected by western blot analysis using antibodies from Santa Cruz and Abcam (Cambridge, MA, USA), as described previously.^11^ Lentiviral GFP control was also used to monitor the infection efficiency.

### Recombinant Jagged1-coated culture plates

The human JAG1 recombinant protein (Enzo Life Sciences, Farmingdale, NY, USA) contains the signal peptide and extracellular domain of JAG1 fused at the C terminus to the Fc portion of human IgG. JAG1-coated plates were generated using the protocol previously described.^12^ Briefly, culture plates (Nunc, polystyrene) were coated with anti-human IgG (10 mg/ml) (Sigma-Aldrich, St. Louis, MO, USA) in PBS at 4 °C overnight, and then incubated in a solution containing recombinant JAG1 protein (10 mg/ml) at 4 °C overnight. The same concentrations of human IgG were used to coat the plates and the controls. All P2 MSCs were cultured on JAG1- and IgG-coated plates for an additional passage (3–5 days) prior to harvest at days 1 and 5 after forming cell sheets, respectively, for the following assays.

### Proliferation and apoptotic assays

P2 MSCs were seeded at 5000 cell/cm^2^ cells in six-well plates (Nunc, polystyrene). At day 1 and day 5 after formation of the cell sheets, the cells were harvested for flow cytometry and proliferation and apoptosis assays. For the proliferation assay, cells were exposed to BrdU-labeling reagent (Roche, Basel, Switzerland) for 6 h, followed by incubation with FixDenat buffer for 30 min, and detection with an anti-BrdU-POD working solution. Absorbance values were measured by a multi-mode microplate reader (BioTek Instruments, Winooski, VT, USA) at 450 nm. The percentage of apoptotic cells was determined using Apo-ONE homogeneous Caspase-3/7 assay (Promega Biosciences, San Luis Obispo, CA, USA). Absorbance values were measured by a multi-mode microplate reader at 530 nm, as described before.^12^

### Flow cytometry analysis

The MSC phenotype was evaluated by the expression of negative marker CD45-APC (BD Biosciences Pharmingen, San Diego, CA) and positive marker CD105-APC (BD Biosciences Pharmingen, San Diego, CA, USA). P2 Cells in sheet culture were incubated for 30 min at room temperature with antibodies, and flow cytometry was performed on a LSR-II flow cytometer (Beckton Dickson). Isotype-matched IgG-APC controls were included and used to set the electronic gates on the flow cytometer. Cell cycle distribution of MSCs from 1-day and 5-day sheet cultures was determined by flow cytometric analysis after labeling with FITC-conjugated anti–Ki-67 (clone MIB-1; Immunotech, Westbrook, ME, USA) and 5 *μ*g/ml 7-aminoactinomycin-D ([7-AAD] Sigma). All the data were analyzed using FlowJo software (Tree Star).

### MSC differentiation assay

MSC spontaneous differentiation assays were performed using standard stem cell growth medium. MSCs were cultured at 5000 cells/cm^2^ to form the cell sheet on standard culture dishes (Nunc, polystyrene) and harvested at days 1 and 5. RNA isolation and ALP staining were performed as previously described.^12^

### Senescence-associated-*β*-galactosidase assay

The senescence-associated-*β*-galactosidase activity was detected using the SA-*β*-gal staining kit (Cell Signaling Technology, Boston, MA, USA) according to the manufacturer's recommendations, except that citric acid/sodium phosphate buffered-staining solution (pH 6.0) was used, as described earlier.^17^ Cells were photographed using an EVOS phase-contrast microscope (Advanced Microscopy Group, Bothell, WA, USA). SA-*β*-gal-positive cells were counted in five randomly selected fields of view to determine the percentage of *β*-gal+ cells (>400 cells were counted).

### Real time RT-PCR

DNA was synthesized from 1 *μ*g total RNA using the SuperScript III reverse transcriptase kit (Invitrogen, Carlsbad, CA, USA) in a final volume of 20 *μ*l. Primers were designed with the IDT SCI primer design tool (Integrated DNA Technologies, San Diego, California, USA). RT-PCR experiments were performed with a Bio-Rad C1000 thermal cycler (Bio-Rad, Hercules, CA, USA) in triplicate. The sequences for each primer pair were as follows: Cyclin D1(CCND1): forward, 5′-ATGGAACATCAGCTGCTGT-3′, and reverse, 5′-TCAGATGTCCACATCCCGC-3′ p21: forward, 5′-GCCTGGACTGTTTTCTCTCG-3′, and reverse, 5′-ATTCAGCATTGTGGGAGGAG-3′ p16: forward, 5′-CACGGGTCGGGTGAGAGT-3′, and reverse, 5′-CCCAACGCACCGAATAGTTAC-3′ alkaline phosphatase (ALP): forward, 5′-GGGCATTGTGACTACCACTC-3′, and reverse, 5′-AGTCAGGTTGTTCCGATTCA-3′ Hes1: forward, 5′- TTCCTCCTCCCCGGTGGCTG-3′, and reverse, 5′-TGCCCTTCGCCTCTTCTCCA-3′ *β*-actin: forward, 5′-ACCACAGTCCATGCCATCAC-3′, and reverse, 5′-TCCACCACCC TGTTGCTGTA-3′. The relative expression level of target genes was normalized with geNorm software (Primer Design Ltd) using *β*-actin gene as a reference to determine the normalization factor.^11^

### MSC sheets transplantation

Regular cultured 5-day MSC sheets and Notch activation cultured 5-day MSC sheets were generated using lgG or JAG1 coated temperature-responsive plates (Nunc UpCell, Thermo Scientific, Cat. 174901). Total of 36 eight-week-old male C57BL/6 J mice were randomly divided into three groups. Following the previously described surgical procedure,^7^ a 4-mm mid-diaphyseal segment bone defect was made, and a 4-mm long allograft wrapped with lgG/5-day sheet or JAG1/ 5-day sheet was then inserted into the segmental defect and stabilized using a 26-gauge metal pin placed through the intramedullary marrow cavity. Allograft alone was used as the control. Mice were killed at six weeks post-surgery and samples processed for X-ray, Micro-CT bone imaging analyses, biomechanical testing, and histological evaluation with Alcian Blue/Hematoxylin/Orange-G (AB/H/OG) staining as described in our published studies.^7^

### Statistical analysis

All experiments were repeated independently at least three times. All data were presented as mean±S.D. Statistical significance among the groups was assessed with one-way ANOVA. The level of significance was *P*<0.05.

## References

[bib1] Bianco P, Cao X, Frenette PS, Mao JJ, Robey PG, Simmons PJ et al. The meaning, the sense and the significance: translating the science of mesenchymal stem cells into medicine. Nat Med 2013; 19: 35–42. 23296015 10.1038/nm.3028 PMC3998103

[bib2] Salem HK, Thiemermann C. Mesenchymal stromal cells: current understanding and clinical status. Stem Cells 2010; 28: 585–596. 19967788 10.1002/stem.269 PMC2962904

[bib3] Bruder SP, Kraus KH, Goldberg VM, Kadiyala S. The effect of implants loaded with autologous mesenchymal stem cells on the healing of canine segmental bone defects. J Bone Joint Surg Am 1998; 80: 985–996. 9698003 10.2106/00004623-199807000-00007

[bib4] Hernigou P, Poignard A, Beaujean F, Rouard H. Percutaneous autologous bone-marrow grafting for nonunions. Influence of the number and concentration of progenitor cells. J Bone Joint Surg Am 2005; 87: 1430–1437. 15995108 10.2106/JBJS.D.02215

[bib5] Xie C, Reynolds D, Awad H, Rubery PT, Pelled G, Gazit D et al. Structural bone allograft combined with genetically engineered mesenchymal stem cells as a novel platform for bone tissue engineering. Tissue Eng 2007; 13: 435–445. 17518596 10.1089/ten.2006.0182 PMC12019782

[bib6] Hoffman MD, Benoit DS. Emulating native periosteum cell population and subsequent paracrine factor production to promote tissue engineered periosteum-mediated allograft healing. Biomaterials 2015; 52: 426–440. 25818449 10.1016/j.biomaterials.2015.02.064 PMC4379449

[bib7] Long T, Zhu Z, Awad HA, Schwarz EM, Hilton MJ, Dong Y. The effect of mesenchymal stem cell sheets on structural allograft healing of critical sized femoral defects in mice. Biomaterials 2014; 35: 2752–2759. 24393269 10.1016/j.biomaterials.2013.12.039 PMC3913373

[bib8] Ben-Porath I, Weinberg RA. The signals and pathways activating cellular senescence. Int J Biochem Cell Biol 2005; 37: 961–976. 15743671 10.1016/j.biocel.2004.10.013

[bib9] Fujita K, Mondal AM, Horikawa I, Nguyen GH, Kumamoto K, Sohn JJ et al. p53 isoforms Delta133p53 and p53beta are endogenous regulators of replicative cellular senescence. Nat Cell Biol 2009; 11: 1135–1142. 19701195 10.1038/ncb1928 PMC2802853

[bib10] Burdon TJ, Paul A, Noiseux N, Prakash S, Shum-Tim D. Bone marrow stem cell derived paracrine factors for regenerative medicine: current perspectives and therapeutic potential. Bone Marrow Res 2011; 2011: 207326. 22046556 10.1155/2011/207326 PMC3195349

[bib11] Dong Y, Jesse AM, Kohn A, Gunnell LM, Honjo T, Zuscik MJ et al. RBPjkappa-dependent Notch signaling regulates mesenchymal progenitor cell proliferation and differentiation during skeletal development. Development 2010; 137: 1461–1471. 20335360 10.1242/dev.042911 PMC2853848

[bib12] Dong Y, Long T, Wang C, Mirando AJ, Chen J, O'Keefe RJ et al. NOTCH-mediated maintenance and expansion of human bone marrow stromal/stem cells: a technology designed for orthopedic regenerative medicine. Stem Cells Transl Med 2014; 3: 1456–1466. 25368376 10.5966/sctm.2014-0034 PMC4250205

[bib13] Iso T, Kedes L, Hamamori Y. HES and HERP families: multiple effectors of the Notch signaling pathway. Journal of Cell Physiol 2003; 194: 237–255. 12548545 10.1002/jcp.10208

[bib14] Williams R, Nelson L, Dowthwaite GP, Evans DJ, Archer CW. Notch receptor and Notch ligand expression in developing avian cartilage. J Anatomy 2009; 215: 159–169. 10.1111/j.1469-7580.2009.01089.x PMC2740963 19490397

[bib15] Kageyama R, Niwa Y, Shimojo H, Kobayashi T, Ohtsuka T. Ultradian oscillations in Notch signaling regulate dynamic biological events. Curr Top Dev Biol 2010; 92: 311–331. 20816400 10.1016/S0070-2153(10)92010-3

[bib16] Engin F, Yao Z, Yang T, Zhou G, Bertin T, Jiang MM et al. Dimorphic effects of Notch signaling in bone homeostasis. Nat Med 2008; 14: 299–305. 18297084 10.1038/nm1712 PMC2671578

[bib17] Wagner W, Horn P, Castoldi M, Diehlmann A, Bork S, Saffrich R et al. Replicative senescence of mesenchymal stem cells: a continuous and organized process. PLOS ONE 2008; 3: e2213. 18493317 10.1371/journal.pone.0002213 PMC2374903

[bib18] Stenderup K, Justesen J, Clausen C, Kassem M. Aging is associated with decreased maximal life span and accelerated senescence of bone marrow stromal cells. Bone 2003; 33: 919–926. 14678851 10.1016/j.bone.2003.07.005

[bib19] Cheung TH, Rando TA. Molecular regulation of stem cell quiescence. Nat Rev Mol Cell Biol 2013; 14: 329–340. 23698583 10.1038/nrm3591 PMC3808888

[bib20] Shimizu T, Sekine H, Isoi Y, Yamato M, Kikuchi A, Okano T. Long-term survival and growth of pulsatile myocardial tissue grafts engineered by the layering of cardiomyocyte sheets. Tissue Eng 2006; 12: 499–507. 16579683 10.1089/ten.2006.12.499

[bib21] Matsuura K, Masuda S, Shimizu T. Cell sheet-based cardiac tissue engineering. Anat Record 2014; 297: 65–72. 10.1002/ar.22834 24343911

[bib22] Bellayr IH, Catalano JG, Lababidi S, Yang AX, Lo Surdo JL, Bauer SR et al. Gene markers of cellular aging in human multipotent stromal cells in culture. Stem Cell Res Ther 2014; 5: 59. 24780490 10.1186/scrt448 PMC4055144

[bib23] Campisi J. Senescent cells, tumor suppression, and organismal aging: good citizens, bad neighbors. Cell 2005; 120: 513–522. 15734683 10.1016/j.cell.2005.02.003

[bib24] Baxter MA, Wynn RF, Jowitt SN, Wraith JE, Fairbairn LJ, Bellantuono I. Study of telomere length reveals rapid aging of human marrow stromal cells following *in vitro* expansion. Stem Cells 2004; 22: 675–682. 15342932 10.1634/stemcells.22-5-675

[bib25] Wang Z, Liu Y, Takahashi M, Van Hook K, Kampa-Schittenhelm KM, Sheppard BC et al. N terminus of ASPP2 binds to Ras and enhances Ras/Raf/MEK/ERK activation to promote oncogene-induced senescence. Proc Natl Acad Sci USA 2013; 110: 312–317. 23248303 10.1073/pnas.1201514110 PMC3538245

[bib26] Rayess H, Wang MB, Srivatsan ES. Cellular senescence and tumor suppressor gene p16. Int J Cancer 2012; 130: 1715–1725. 22025288 10.1002/ijc.27316 PMC3288293

[bib27] Janzen V, Forkert R, Fleming HE, Saito Y, Waring MT, Dombkowski DM et al. Stem-cell ageing modified by the cyclin-dependent kinase inhibitor p16INK4a. Nature 2006; 443: 421–426. 16957735 10.1038/nature05159

[bib28] Kobayashi T, Kageyama R. Hes1 regulates embryonic stem cell differentiation by suppressing Notch signaling. Genes Cells: Devoted Mol Cell Mech 2010; 15: 689–698. 10.1111/j.1365-2443.2010.01413.x PMC2916211 20545770

